# COVID-19 transmission in Africa: estimating the role of meteorological factors

**DOI:** 10.1016/j.heliyon.2022.e10901

**Published:** 2022-10-02

**Authors:** Montasir Ahmed Osman

**Affiliations:** Mathematics Department, Faculty of Education, University of Kassala, Kassala, Sudan

**Keywords:** Coronavirus, Generalized linear models, Poisson regression, Weather conditions

## Abstract

Climate variables play a critical role in COVID-19’s spread. Therefore, this research aims to analyze the effect of average temperature and relative humidity on the propagation of COVID-19 in Africa's first four affected countries (South Africa, Morocco, Tunisia, and Ethiopia). As a result, policymakers should develop effective COVID-19 spread control strategies. For each country, using daily data of confirmed cases and weather variables from May 1, 2020, to April 30, 2021, generalized linear models (Poisson regression) and general linear models were estimated. According to the findings, the rising average temperature causes COVID-19 daily new cases to increase in South Africa and Ethiopia while decreasing in Morocco and Tunisia. However, in Tunisia, the relative humidity and daily new cases of COVID-19 are positively correlated, while in the other three countries, they are negatively associated.

## 1 Introduction

The Coronavirus disease 2019 (COVID-19), which was first discovered in Wuhan, China, in December 2019, is a severe global pandemic in the third decade of the twenty-first century (WHO, 2022a, WHO, 2022b). The COVID-19 seriousness comes from two sides; the swiftness of the pandemic dissemination within and between countries and the high mortality rate of affected people. As of August 8, 2022, more than 581 million people have been affected globally, and more than 6 million have lost their lives (WHO, 2022a, WHO, 2022b).

Meteorological variables, such as temperature and humidity, could play a vital role in the coronavirus’s survival in the climate. Therefore, the function of the weather factors could be significant in COVID-19 transmission (McClymont and Hu, 2021). So, much research has been done to discover the association between the COVID-19 spread and the environmental factors worldwide (Uzair et al., 2022; Weaver et al., 2022; Zhang et al., 2022). Moreover, the findings of these studies are not constant; while a negative link between COVID-19 transmission was reported in some papers, a positive correlation was detected by others.

The relationship between COVID-19 and atmospheric temperature has been extensively researched worldwide. While some studies found negative associations, others found positive connections or no evidence of a correlation. For instance, a 30-day of data study in Spain found that temperature is significantly negatively correlated with daily new cases of COVID-19 (Paez et al., 2021). Subsequently, based on 41 days of data, rising temperatures are linked to fewer daily confirmed COVID-19 cases in China (Shi et al., 2020). In addition, based on data from 38 days, the significance of the negative correlation between temperature and COVID-19 daily confirmed cases is stated in the United States (Chien and Chen, 2020). Using 95 days of data from Saudi Arabia, a researcher highlighted a negative relationship between daily confirmed cases and temperature (Alkhowailed et al., 2020). In addition, based on 31 days of data, the decline in COVID-19 transmission is linked to an increase in the mean temperature in Africa (Adekunle et al., 2020). On the other hand, other studies have found a positive link between confirmed COVID-19 cases and temperature. Based on 66 days of data, researchers discovered a positive association between COVID-19 cases and the maximum temperature in Norway (Menebo, 2020). Similar findings were found in India (Meraj et al., 2021), Singapore (Pani et al., 2020), and China (Xie and Zhu, 2020). Third, other papers demonstrated insignificant correlation between COVID-19 spread and atmospheric temperature in Spain (Briz-Redón and Serrano-Aroca, 2020) and Canada (To et al., 2021).

Accordingly, the relationship between COVID-19 spread and humidity shifts from negative to positive. In Saudi Arabia (Alkhowailed et al., 2020), Spain (Paez et al., 2021), and Australia (Ward et al., 2020), a significant negative correlation between COVID-19 confirmed new cases and humidity were reported. on the other hand, a study in the United States revealed a significant positive relationship between COVID-19 new cases and humidity (Chien and Chen, 2020). Other studies in Africa (Adekunle et al., 2020) and Indonesia (Tosepu et al., 2020) found a negligible, neither positive nor negative, link between COVID-19 spread and humidity.

Overall, according to the above discussion, the effect of weather factors on COVID-19 incidence is still vacillating. In addition, there is a shortage of COVID-19 transmission studies in African countries (Diop et al., 2020). Therefore, more research on this impact is motivated, particularly in African countries. So, this research aimed to ascertain the association between the daily new confirmed cases of COVID-19 and meteorological factors, including the daily average temperature and relative humidity, in the first four affected countries in Africa (South Africa, Morocco, Tunisia, and Ethiopia). Depending on data for 365 days (from May 1, 2020, to April 30, 2021), the study estimates the effect of independent variables on the target variable using generalized linear models (Poisson regression) as well as linear regression. To the best of my knowledge, this research was based on the most extensive data series, in terms of duration of the data series. While the full-year data used in this study, other papers utilized information for 13 days (Notari, 2021; Oliveiros et al., 2020), 39 days (Pawar et al., 2020) and 55 days (Cambaza et al., 2020). Therefore, this variety of data may be one of the work's strengths. Additionally, this article is unique in that it examines the first four African nations affected by COVID-19.

## 2 Materials and methods

### 2.1 Data of the research

The daily COVID-19 incidences, mean temperature (C), and relative humidity (percentage), as meteorological parameters, for the top four countries in Africa with the highest COVID-19 incidence (South Africa, Morocco, Tunisia, and Ethiopia) as of April 30, 2021, were utilized in this study.

According to the World Health Organization (WHO) statistics, over 149 million people are suffering from COVID-19 as of April 30, 2021, universal (WHO, 2022a, WHO, 2022b). More than three million of that figure are in Africa. The four most affected African countries are South Africa, Morocco, Tunisia, and Ethiopia, with cumulative cases 1,578,450, 510,465, 305,313, and 255,288 for each country, respectively, as of April 30, 2021. The COVID-19 data are collected for the four mentioned countries as the number of new cases per day for one year from May 1, 2020, to April 30, 2021, from the Data Hub website (DataHub, 2022).

As weather factors, the average temperature (C) and relative humidity (%) are used as interpreter variables in this study. Both variables were collected per day from May 1, 2020, to April 30, 2021, in the capitals of the studied countries. The paper depends on NASA website data according to the country capital coordinates (latitude, longitude), South Africa (−33.928992, 18.417396), Morocco (34.022405, −6.834543), Tunisia (36.806389, 10.181667), and Ethiopia (9.0107934, 38.7612525) (NASA, 2021).

### 2.2 Methods

In this research, graphical display, descriptive statistics, correlation, simple and multiple linear regression, in addition to simple and multiple Poisson regression, are utilized.

Firstly, the data were visualized graphically to explore the possible association between the dependent variable (daily COVID-19 cases) and the weather factors, explorer variables, including the temperature and relative humidity. Furthermore, this data representation could be an efficient way to explore the data shape patterns. Specifically, the normality or non-normality of data distribution and the linearity or non-linearity of curves will be detected easily.

Then, descriptive statistics, which are considered the best, most precise, and understandable way to summarize a dataset, are used to display and show the characteristics of data sets. Moreover, these statistics are helpful for preliminary information and potential relationship highlighting within dataset variables. The measures implemented for these purposes are mean, standard deviation, median, minimum value, maximum value, first quartile, and third quartile.

In addition, the correlation between dataset variables was computed and visualized using R software (STHDA, 2022). Correlation is functional and commonly used in research that tests the relationships between COVID-19 infections and weather conditions. For instance, the likely impact of atmospheric pressure and temperature on COVID-19 daily confirmed cases is examined in some African countries (Cambaza et al., 2020).

Next, simple and multiple regression models (Hoffmann, 2021; Montgomery et al., 2013; Yan, 2009) are estimated. Generally, many studies have implemented linear regression to explore the association between COVID-19 cases and the weather conditions (Nguimkeu and Tadadjeu, 2021; Pawar et al., 2020; Zhang et al., 2021). Of course, in our models, while the dependent variable was the COVID-19 case per day for each studied country, the independent variables were the daily average of temperature (C) and relative humidity.

Finally, this paper used simple and multiple Poisson regression to capture the potential relationship between COVID-19 infections and weather conditions such as average temperature and relative humidity. The Poisson regression models may be recommended when the target variable is countable and skewed (Cameron and Trivedi, 2013; Consul and Famoye, 1992; Myers et al., 2010). Thus, the Poisson regression approach is often used in COVID-19 investigations. In some European countries, this approach is used to investigate the association between daily COVID-19 infections and climatic parameters such as temperature and absolute humidity (Gharoie Ahangar et al., 2020).

In this section, the results of the study will be shown. These findings include descriptive statistics and analytical outcomes from linear and Poisson regression models. While the basic statistics are shown in Table 1, the estimated model results are illustrated in Tables 2, 3, 4, and 5.

**Table 1:** Descriptive statistics of the study variables.

Country	Statistic	Daily COVID-19 Cases	Average Temperature	Relative Humidity
South Africa	Minimum	236	10.24	46.33
	1st Quartile	1371.25	14.745	71.74
	Median	2184.5	16.735	76.25
	3st Quartile	6517.75	18.6925	82.09
	Maximum	21,980	22.64	92.71
	Mean	4412	17	76
	St.D.	4630	3	8
Morocco	Minimum	24	11.66	43.02
	1st Quartile	338.5	16.275	70.8175
	Median	859	19.28	74.51
	3st Quartile	2359.25	22.775	79.03
	Maximum	6195	29.03	91.72
	Mean	1471	20	75
	St.D.	1471	4	7
Tunisia	Minimum	0	7.82	32.91
	1st Quartile	461.25	13.65	60.61
	Median	1086.5	18.81	67.89
	3st Quartile	1677.5	24.46	74.455
	Maximum	5752	33.45	90.05
	Mean	1163	19	67
	St.D.	929	6	11
Ethiopia	Minimum	0	10.81	16.57
	1st Quartile	364	14.04	53.2225
	Median	583	15.34	67.08
	3st Quartile	982	16.71	79.8725
	Maximum	2372	19.99	91.53
	Mean	748	15	65
	St.D.	566	2	18

**Table 2:** Estimated simple linear regression models results.

Country	Term	Average Temperature				Relative Humidity			
		Estimate	Std. error	t-value	p-value	Estimate	Std. error	t-value	p-value
South Africa	Intercept	7.929	0.36	22.28	0.000	8.200	0.54	15.10	0.000
	Slope	-0.003	0.02	-0.15	0.884	-0.004	0.01	-0.60	0.600
Morocco	Intercept	7.337	0.35	21.11	0.000	4.487	0.69	6.48	0.000
	Slope	-0.039	0.02	-2.25	0.030	0.028	0.01	3.02	0.003
Tunisia	Intercept	9.811	0.40	24.3	0.000	-2.577	0.87	-2.97	0.003
	Slope	-0.258	0.02	-12.8	0.000	0.111	0.01	8.74	0.000
Ethiopia	Intercept	6.745	0.69	9.76	0.000	7.292	0.302	24.129	0.000
	Slope	-0.049	0.04	-1.10	0.300	-0.020	0.004	-4.449	0.000

**Table 3:** Estimated multiple linear regression models results.

Country	Term	Average Temperature				Relative Humidity			
		Estimate	Std. error	z-value	p-value	Estimate	Std. error	t-value	p-value
South Africa	Intercept	7.633	0.0056	1363.42	0.000	9.147	0.0082	1121.39	0.000
	Slope	0.044	0.0003	134.16	0.000	-0.010	0.0001	-95.31	0.000
Morocco	Intercept	7.567	0.0072	1056.42	0.000	7.051	0.0146	483.97	0.000
	Slope	-0.017	0.0004	-46.78	0.000	0.002	0.0002	12.78	0.000
Tunisia	Intercept	8.782	0.0059	1495.67	0.000	3.599	0.0136	265.54	0.000
	Slope	-0.120	0.0004	-327.97	0.000	0.045	0.0002	241.27	0.000
Ethiopia	Intercept	4.238	0.018	237.01	0.000	7.465	0.0067	1121.12	0.000
	Slope	0.149	0.001	132.62	0.000	-0.014	0.0001	-136.90	0.000

**Table 4:** Estimated simple Poisson regression models results.

Country	Term	Estimate	Std. error	t-value	p-value
South Africa	Intercept	8.372	0.756	11.069	0.000
	Temp	-0.007	0.022	-0.327	0.744
	RH	-0.005	0.007	-0.665	0.507
Morocco	Intercept	5.268	0.95	5.54	0.000
	Temp	-0.023	0.02	-1.20	0.232
	RH	0.023	0.01	2.34	0.020
Tunisia	Intercept	8.207	1.49	5.50	0.000
	Temp	-0.237	0.03	-8.53	0.000
	RH	0.018	0.02	1.12	0.265
Ethiopia	Intercept	8.941	0.809	11.057	0.000
	Temp	-0.098	0.045	-2.197	0.029
	RH	-0.022	0.005	-4.853	0.000

**Table 5:** Estimated multiple Poisson regression models results.

Country	Term	Estimate	Std. error	z-value	p-value
South Africa	Intercept	8.223	0.0118	699.7	0.000
	Temp	0.038	0.0003	110.3	0.000
	RH	-0.006	0.0001	-56.9	0.000
Morocco	Intercept	7.659	0.0196	389.9	0.000
	Temp	-0.018	0.0004	-45.5	0.000
	RH	-0.001	0.0002	-5.0	0.000
Tunisia	Intercept	7.680	0.0201	382.5	0.000
	Temp	-0.110	0.0004	-263.5	0.000
	RH	0.013	0.0002	57.8	0.000
Ethiopia	Intercept	5.547	0.0212	261.6	0.000
	Temp	0.110	0.0011	97.1	0.000
	RH	-0.011	0.0001	-101.0	0.000

### 2.3 Descriptive statistics

Table 1 shows descriptive statistics for the dataset variables (daily COVID-19 confirmed cases, average temperature, and relative humidity) for the targeted countries (South Africa, Morocco, Tunisia, and Ethiopia) over a year (365 days) from May 1, 2020, to April 30, 2021. The minimum, first quartile, median, third quartile, maximum, mean, and standard deviation are the parameters in Table 1.

According to Table 1, South Africa has the highest minimum value of COVID-19 confirmed cases per day (236), while both Tunisia and Ethiopia have the smallest daily infections (zero). Considering the first quartile, South Africa also has the maximum value (1371) of new daily cases compared to Morocco, which has the lowest value (339). Again, South Africa recorded the maximum median of daily COVID-9 cases, followed by Tunisia, Morocco, and Ethiopia, respectively. Regarding the third quartile and the maximum value of daily COVID-19 new cases, the decreasing order of these countries is South Africa, Morocco, Tunisia, and Ethiopia. Likewise, in the study period, while the greatest mean for the daily new confirmed cases of COVID-19 was reported in South Africa (4412), the second, third, and fourth means were registered, correspondingly, in Morocco (1471), Tunisia (1163) and Ethiopia (748). As well as the above measures, the highest standard deviation of daily COVID-19 infections was shown in South Africa (4630), followed by Morocco (1471), then Tunisia (929), and Ethiopia (566). South Africa has the highest numbers for the four studied countries, whereas Ethiopia has the smallest one regarding the daily COVID-19 cases.

Accordingly, the order of the mentioned countries depends on average temperature (C) is Tunisia (7.82), South Africa (10.24), Ethiopia (10.81), and Morocco (11.66). However, regarding the maximum values of average temperature, the decreasing order of these countries is Tunisia (33.45), Morocco (29.03), South Africa (22.64), and Ethiopia (13.99). In addition, depending on the mean and the standard deviation (Mean/St.Dev) of daily average temperature, Ethiopia is the coldest one (15/2), followed by South Africa (17/3), then Tunisia (19/9) and Morocco (20/4).

Furthermore, as shown in Table 1, the biggest value of relative humidity (%) are reported in South Africa, followed by Morocco, Tunisia, and Ethiopia concerning mean, median, and minimum values over the studied year. In contrast, according to the spread measure (standard deviation) of the relative humidity, the arrangement of these countries, from the smallest to the largest is South Africa, Morocco, Tunisia, and Ethiopia, respectively.

### 2.4 Data visualization

The study datasets are graphically visualized in this section to identify the variable patterns, trends, and distributions shapes. The visualization includes displaying variables in histograms, plots for single variables, plots for two variables and curves, and correlation.

**Figure 1:**
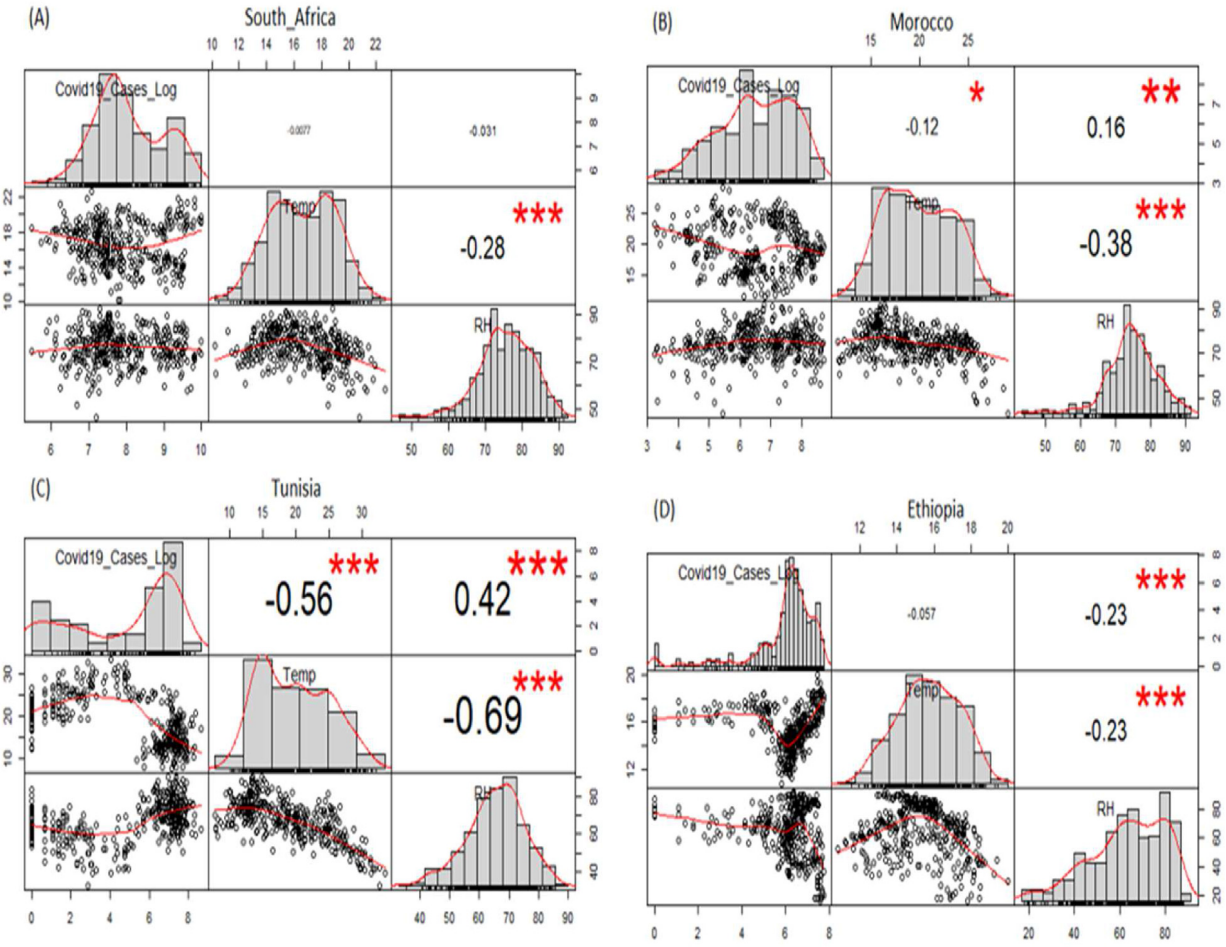
Correlation matrix of COVID-19 cases (log), average temperature and relative humidity.

The correlation matrix of the study dataset variables for the chosen countries (South Africa, Morocco, Tunisia, and Ethiopia) is shown in Figure 1. The diagonal cells of this matrix are used to exhibit the histograms of variables, while the cells above the diagonal are used to display the correlation coefficients between each pair of variables and cells lower the diagonal are utilized to show the scatter plot of each combination variables. These coefficient values print in the graph with font sizes related to their values. The highest correlation coefficient prints in the largest font size and vice versa. The variables in this graph are the logarithm (log) of daily new COVID-19 reported cases, daily average temperature, and relative humidity for each country. In South Africa, as shown in Figure 1-a, the diagonal represented the histograms of confirmed cases, daily average temperature and relative humidity from upper to lower, respectively. Regarding to correlation coefficients, the negative correlation between the daily average temperature and relative humidity is statistically significant (r = −0.28, p-value < 0.001), the third cell in the second row in Figure 1-a. Although, the associations between the logarithm of daily new confirmed COVID-19 cases and the daily average temperature and relative humidity are insignificant (the second and third cells in the first row, Figure 1-a). In Morocco, however, as shown in Figure 1-b, each variable is significantly correlated with the other two variables. Person's correlation coefficients for daily new confirmed cases of COVID-19 are −0.12 (p-value < 0.05) and 0.16 (p-value < 0.01), respectively, with the daily average temperature and relative humidity, while the average temperature and relative humidity have a correlation coefficient of −0.38 (p-value < 0.001). In Tunisia, as shown in Figure 1-c, each combination of two variables has a significant correlation coefficient. Conversely, in Ethiopia, while relative humidity is significantly correlated with two other variables, the daily average temperature is not significantly correlated with COVID-19 incidence, as shown in Figure 1-d.

**Figure 2:**
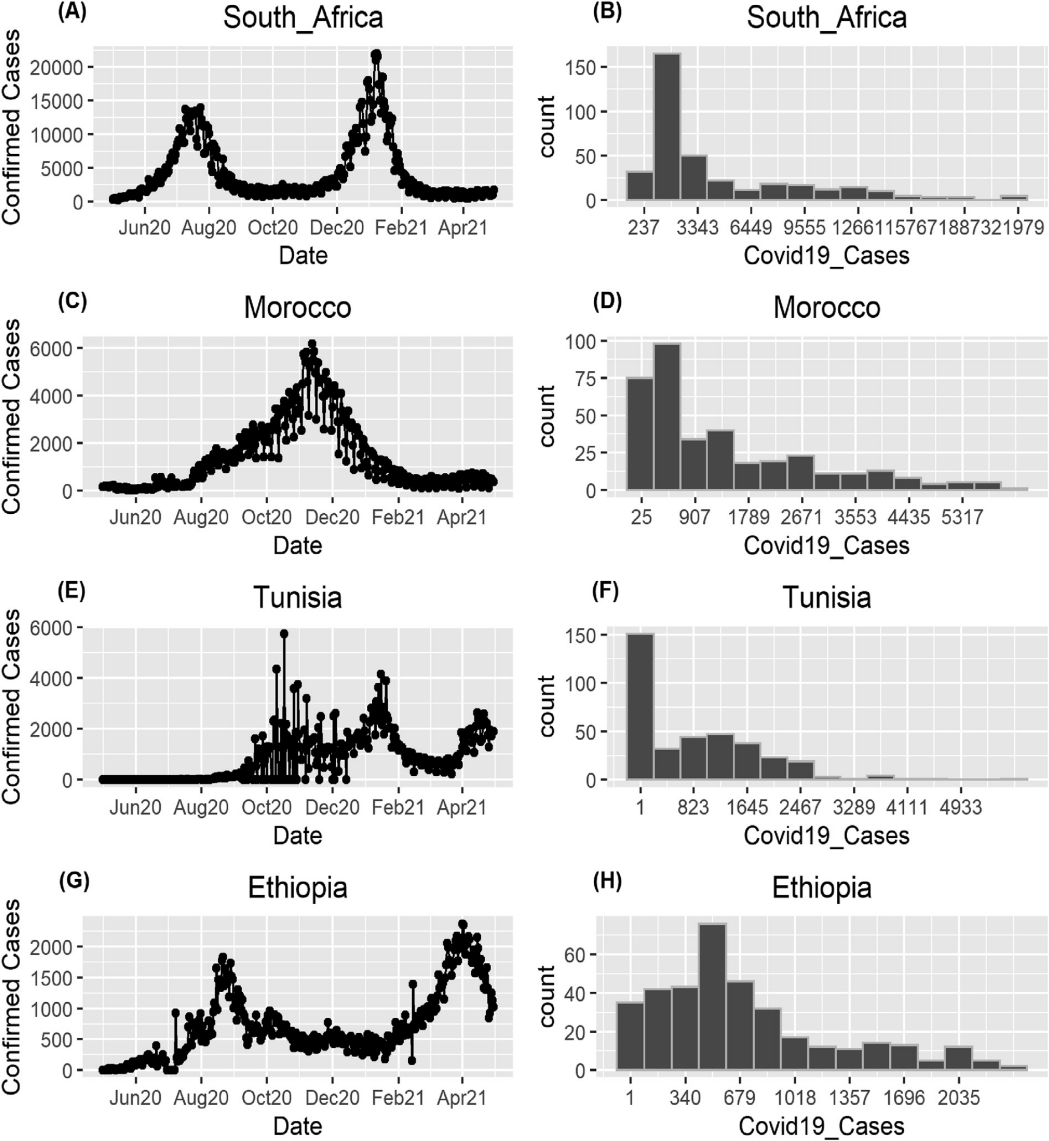
COVID-19 daily confirmed cases curves and histograms.

Figure 2 displays the curves and histograms of the daily new confirmed cases of COVID-19 for the four studied countries (South Africa, Morocco, Tunisia, and Ethiopia) during the study period (from May 1, 2020, to April 30, 2021). In curves, while the horizon axis showed the date (from May 1, 2020 to April 30, 2021), the vertical axis presented the number of confirmed cases per day. In the cases of histograms, the horizon axis exhibits the number of daily new COVID-19 confirmed cases when the vertical one presented the count of these numbers. Firstly, the curves in Figure 2-A and 2-G reveal that the COVID-19 incidence has two peaks in South Africa and Ethiopia. Specifically, the smallest one in July 2020 and the biggest one in January 2021 in South Africa when in Ethiopia, the smallest one in September 2020, and the highest one in April 2021. Conversely, the curve of confirmed cases has one peak in November 2020 in Morocco (Figure 2-C) and in January 2021 in Tunisia (Figure 2-E). Next, the histograms show the daily new confirmed cases of COVID-19 that are right-skewed in the mentioned countries, all histograms showed high frequencies on the left (small values) and low frequencies on the right (high values), according to Figure 2-B, 2-D, 2-F, and 2-H.

**Figure 3:**
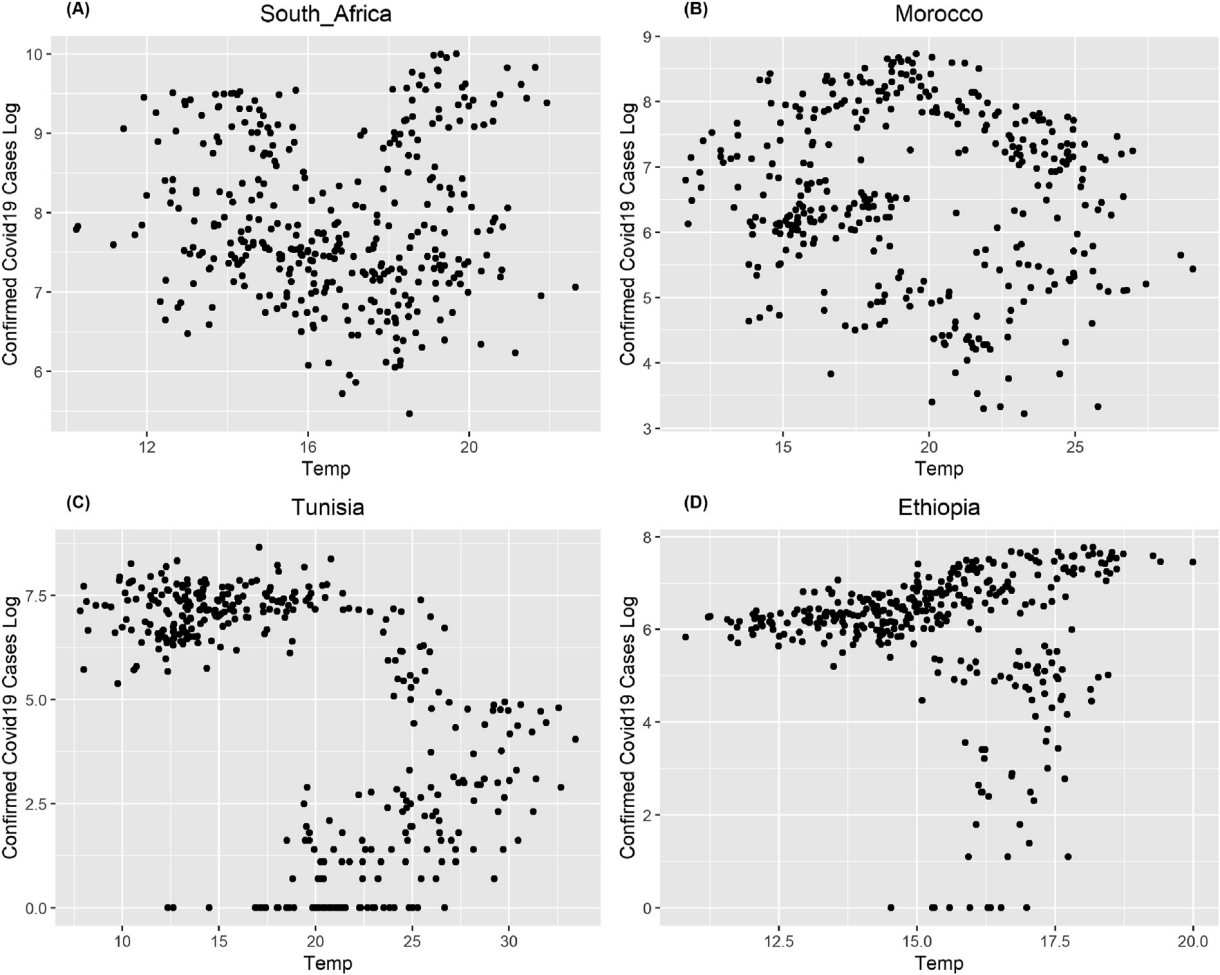
Scatter plot of COVID-19 cases (log) and average temperature.

Figure 3 shows the scatter plots of the daily new confirmed cases of COVID-19 (transfer to logarithms) and the daily average temperature in the studied countries to discover the potential associations between variables. First, according to Figure 3-C, there is a noticeable association between the confirmed cases and the temperature in Tunisia. Furthermore, more COVID-19 cases are reported when the temperature is less than 18 (C). Then, as shown in Figure 3-B, aquiet relationship between the COVID-19 infections and the daily temperature could be remarkable in Morocco, where most COVID-19 cases are registered at a temperature between 17 and 23 (C). Finally, according to Figure 3-A and 3-D, the distribution of scatter plots points exhibits no remarkable links between the COVID-19 infections and the daily average temperature in South Africa and Ethiopia.

**Figure 4:**
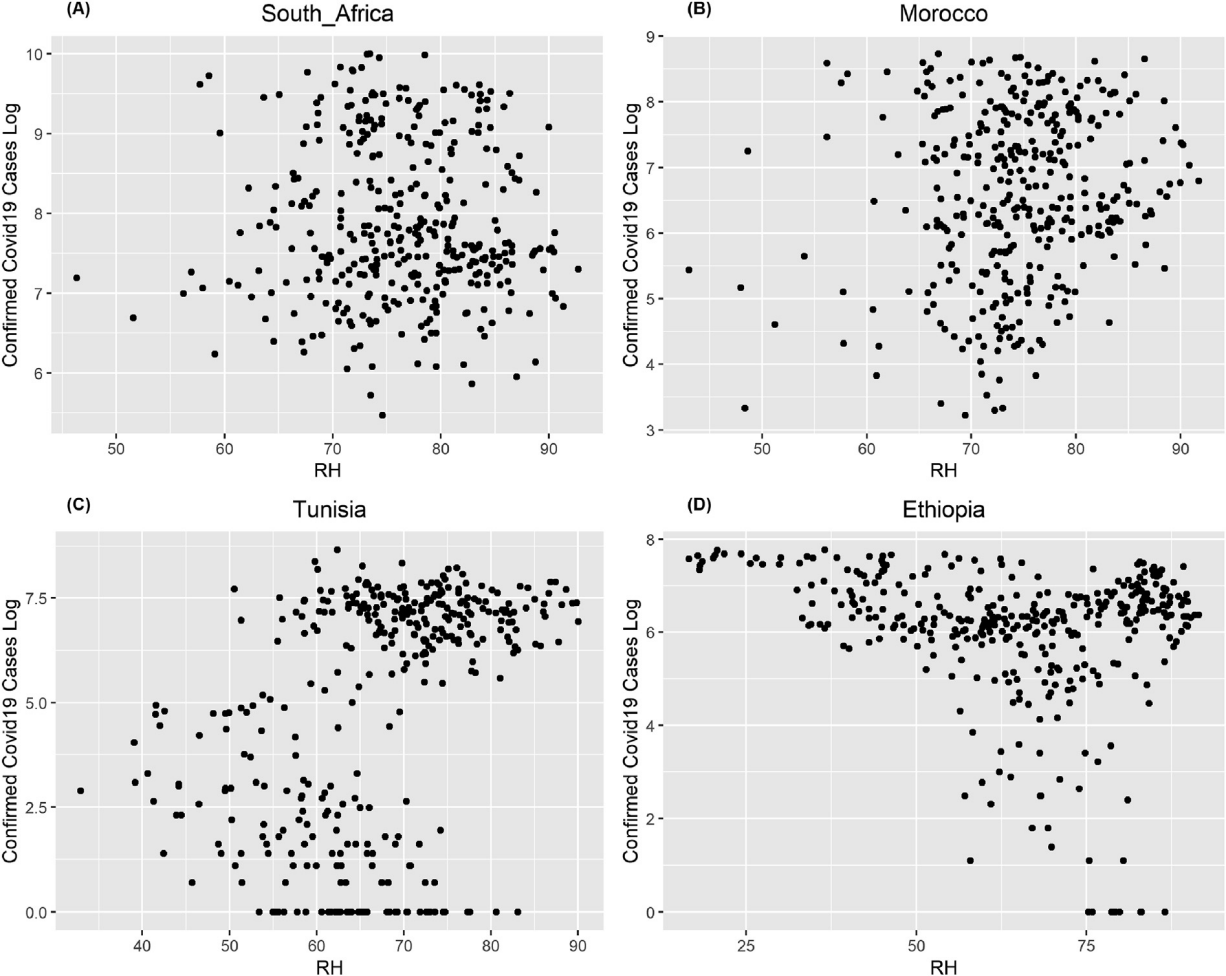
Scatter plot of COVID-19 cases (log) and relative humidity.

Figure 4 displays the scatter plots of the daily new confirmed cases of COVID-19 and the relative humidity in the mentioned countries. This displaying reveals the relative humidity and COVID-19 cases are positively correlated in Tunisia (Figure 4-C) and Morocco (Figure 4-B), negatively correlated in Ethiopia (Figure 4-D), and uncorrelated in South Africa (Figure 4-A).

### 2.5 Estimated linear regression models

The estimated coefficients of simple linear regression models are shown in Table 2. These models investigated the possible links between daily new confirmed COVID-19 cases and meteorological parameters including relative humidity (%) and average temperature (C) in Africa's first four affected countries (South Africa, Morocco, Tunisia, and Ethiopia). The log of daily new confirmed COVID-19 cases plus one (to avoid a log of zero) for each country is the response variable in the models, while the explanatory variables are the daily average temperature and relative humidity.

In general, the estimated simple linear regression results show that the average temperature has a negative effect on daily new confirmed COVID-19 cases in the four countries. Furthermore, the logarithm of daily new confirmed cases of COVID-19 decreases by 0.003 in South Africa, 0.039 in Morocco, 0.258 in Tunisia, and 0.049 in Ethiopia when the daily average temperature rises by 1° (C). In the cases of Morocco and Tunisia, the effect of the regression coefficients is statistically significant at a significance level of 0.05, but in the cases of South Africa and Ethiopia, these coefficients are statistically insignificant at the same significance level.

However, the impact of relative humidity on the daily new confirmed cases of COVID-19 is oscillating between negative and positive from one country to another. To demonstrate, the raising in relative humidity leads to high infections in Morocco and Tunisia, but this rising drives to low cases in South Africa and Ethiopia. Specifically, when the relative humidity rises by 1 (%), the logarithm of daily confirmed cases grows by 0.028 in Morocco and 0.111 in Tunisia. In contrast, when the relative humidity increases by 1 (%), the logarithm of daily confirmed cases declines by 0.004 in South Africa and 0.020 in Ethiopia. Regrading to the significance of the models, except the South Africa model, all the estimated models are statistically significant.

Table 3 demonstrates the outcomes of multiple linear regression models of the daily new confirmed cases of COVID-19 on the daily average temperature and relative humidity. Statistically, multiple regression produces result better than simple regression when the independent variables are significantly correlated. In our case, as the visualization section has revealed, the daily average temperature and relative humidity are significantly associated in the four studied countries. Therefore, the results of the multiple linear regression models could be highly considered.

In general, as the results show, the directions of the relationships are the same in simple linear regression models when the significance of the models and regression slopes have differed. Notably, by controlling the relative humidity, the daily average temperature increasing led to the daily new confirmed cases of COVID-19 decreasing in the four countries (South Africa, Morocco, Tunisia, and Ethiopia). This impact is significant in Tunisia and Ethiopia but insignificant in South Africa and Morocco. In contrast, when the daily average temperature is controlled, when the relative humidity rises, the daily new confirmed cases of COVID-19 reduce in South Africa and Ethiopia but grow in Morocco and Tunisia. These models coefficients are statistically significant in cases of Morocco and Ethiopia and insignificant otherwise.

### 2.6 Estimated Poisson regression models

The estimated simple and multiple Poisson regression models of daily new confirmed COVID-19 cases on the daily average temperature and relative humidity are shown in Tables 4 and 5, respectively. When the target variable is countable and skewed, such as the number of daily new confirmed cases of COVID-19, Poisson regression may be highly recommended (Kim et al., 2021; Odhiambo et al., 2020).

In contrast to linear regression outputs, the effect of daily average temperature on daily new confirmed cases of COVID-19 in the four countries studied is statistically significant (at 0.05 significance level), as shown in Table 4 (South Africa, Morocco, Tunisia, and Ethiopia). Furthermore, in South Africa and Ethiopia, COVID-19 transmission is faster at high average temperatures (the slope is positive). On the contrary, COVID-19 spreads faster in Morocco and Tunisia when the average temperature is low (regression coefficient is negative).

Similarly, all estimated models are statistically significant for the four countries regarding the impact of the relative humidity on the daily new confirmed cases of COVID-19. The regression slope is positive in cases of Morocco and Tunisia and negative otherwise.

According to the outputs in Table 5, all fitted multiple Poisson models are statistically significant. Further, at some relative humidity level, if the daily average temperature increases by 1° (C), the log of daily new confirmed cases of COVID-19 will increase by 0.038, −0.018, −0.110, and 0.110 in South Africa, Morocco, Tunisia, and Morocco respectively. Similarly, if the daily average temperature is controlled, a 1% rise in the relative humidity will decline the log of daily new confirmed cases of COVID-19 by 0.006, 0.001, −0.013, and 0.011 in South Africa, Morocco, Tunisia, and Ethiopia, respectively.

## 3 Discussion

The impact of weather factors, which including daily average temperature and relative humidity, on the spread of COVID-19 in the first four affected African countries (South Africa, Morocco, Tunisia, and Ethiopia), was investigated using linear models and generalized linear models (Poisson regression) in this study. The findings show that the impact of meteorological variables on the COVID-19 transition is still debatable. In other words, each weather factor has a positive or negative impact on COVID-19 transmission in some countries. Hence, it is useful for policy makers to formulate a suitable COVID-19 spread controlling plans. This conclusion is consistent with previous research that has mentioned this variation.

The generated results in Table 5 demonstrate The daily average temperature has a positive effect on the daily new confirmed cases of COVID-19 in South Africa and Ethiopia,. A 1 °C increase in average temperature is associated with a 0.038 rise in log of new confirmed cases in South Africa, given the relative humidity is held constant. In Ethiopia, a 1 °C increase in the average temperature is correlated with a 0.110 increase in the log number of new confirmed cases, assuming that the relative humidity remains constant. In France (Gharoie Ahangar et al., 2020), Norway (Menebo, 2020), India (Meraj et al., 2021), Singapore (Pani et al., 2020), and China (Xie and Zhu, 2020) a parallel findings were pointed. In Morocco and Tunisia, on the other hand, the daily average temperature has a negative impact on the daily new confirmed cases of COVID-19. Under the assumption that the relative humidity stays constant, an increase in the average temperature of 1 °C in Morocco is linked with a decrease in the log number of new confirmed cases of 0.018. Given that the relative humidity is maintained constant, a 1 °C increase in the average temperature is linked to a 0.110 decrease in the log number of new confirmed cases in Tunisia. These findings are comparable to those in Africa (Adekunle et al., 2020), Saudi Arabia (Alkhowailed et al., 2020), the United States (Chien and Chen, 2020), Spain (Gharoie Ahangar et al., 2020), and China (Liu et al., 2020). As a result, making a specific derivation about the effect of temperature on the COVID-19 spread could be difficult.

Similarly, the results of the generalized regression models, in Table 5, reveal the inconsistency of the humidity effect on COVID-19 transmission. In South Africa, Morocco, and Ethiopia, there is a significant negative relationship between daily new confirmed COVID-19 cases and relative humidity, while a significant positive association was demonstrated in Tunisia. Supposing unchanged daily average temperature, a 1% relative humidity increase is associated with 0.006, 0.001, 0.011 and -0.013 reduction in the log of daily new confirmed cases in South Africa, Morocco, Ethiopia and Tunisia, respectively. These findings are similar to those in Spain (Gharoie Ahangar et al., 2020), the world (Zhang et al., 2021), China (Oliveiros et al., 2020), Australia (Ward et al., 2020) and in Italy (Gharoie Ahangar et al., 2020).

## 4 Conclusion

This study examined at the correlations between daily new confirmed COVID-19 cases and daily average temperature and daily relative humidity in Africa’s top four impacted countries. General and generalized linear models are computed for this purpose. The findings show that confirmed cases are inversely connected with average temperature in certain countries and positively associated with average temperature in others. Likewise, while there is a positive correlation between daily new confirmed COVID-19 cases and relative humidity in certain countries, there is a negative association in others. As a result, determining whether the relationship between COVID-19 transmission is positive or negative may be a challenging.

To my best knowledge, regarding the length of the data series, this paper was conducted on the most extended series of data. While this research depends on full-year data (365 days: from May 1, 2020, to April 30, 2021), other papers used data for 13 days (Notari, 2021; Oliveiros et al., 2020), 39 days (Pawar et al., 2020) and 55 days (Cambaza et al., 2020). So, this range of data may be one of the strengths of this work. Furthermore, this paper is singular in the study of the first four COVID-19 affected countries in Africa.

On the other hand, the limitation of this paper is regarding meteorological data collection. While the daily new confirmed cases of COVID-19 were gathered from the whole country, the weather variables data were only collected from the country's capital. Therefore, more studies that analyze COVID-19 cases and weather factors from the same city are recommended.

## Declarations

### Author contribution statement

Montasir Osman: Conceived and designed the experiments; Performed the experiments; Analyzed and interpreted the data; Contributed reagents, materials, analysis tools or data; Wrote the paper.

### Funding statement

This research did not receive any specific grant from funding agencies in the public, commercial, or not-for-profit sectors.

### Data availability statement

Data will be made available on request.

### Declaration of interest’s statement

The authors declare no conflict of interest.

### Additional information

No additional information is available for this paper.
